# Why do consumers respond to eco-labels? The case of Korea

**DOI:** 10.1186/s40064-016-3550-1

**Published:** 2016-11-04

**Authors:** Jung-Ah Hwang, Youkyoung Park, Yeonbae Kim

**Affiliations:** 1 Technology Management, Economics and Policy Program (TEMEP), College of Engineering, Seoul National University, 1 Gwanak-ro, Gwanak-gu, Seoul, 08826 Republic of Korea; 2 Department of Knowledge Service Engineering, KAIST, 291 Haekak-ro, Youseong-gu, Daejeon, 305-701 Republic of Korea

## Abstract

Although eco-labels were introduced with the intention of encouraging eco-friendly purchasing behavior by consumers, they have had little effect on consumers’ purchasing decisions, and therefore a significant gap exists between eco-label awareness and actual purchasing behavior. The aim of this study was to analyze consumer preference, in terms of public and private values, for two types of Korean eco-label that have been administered by the Korean government since 1992. Analyses were based on a structural equation model, employing the theory of reasoned action. Data were collected by survey. The results indicate that although general consumers are highly aware of the publicly valuable information that eco-labels provide, privately valuable information exerts far greater power over their purchasing intentions. Therefore, a supplementary policy that converts public value to private value could promote the purchase of eco-labeled products.

## Background

The introduction of eco-labels, which provide eco-information on products, was expected to encourage consumers to purchase eco-friendly products. Many researchers considered that the use of eco-labels would boost the market for eco-friendly products. However, since its introduction, eco-labeling seems to have barely affected purchasing decisions among consumers (Rex and Baumann 2007). Although consumers express a higher willingness to pay for eco-labeled products, this has not been realized in practice. The impact of eco-labels has been much lower than expected, and little increase has been seen in the market share of eco-labeled products.

This marks the starting point of our research. Most exist studies on eco-labels and consumer behavior have focused on the behavior of eco-friendly consumers. However, to increase the market share for eco-friendly products, it is important to influence purchasing decisions among general consumers. In other words, research on eco-labels and consumer behavior should focus on the purchasing behavior of general consumers. In this paper, we examine the preferred value of eco-labels among general consumers in South Korea.

The contribution of our research is as follows.

1. We focused on the role of consumers’ perceived private value to investigate consumers’ purchasing intention on eco-labeled products.
2. We analyzed the effect of consumers’ perceived social and private value on consumers’ purchasing intention and compared both effects in Korea, using survey data.
3. We found that consumers’ perceived private value is more effective than their perceived social value in encouraging consumers’ purchasing intention.

In “Eco-labels in Korea” section, we introduce two types of eco-label in Korea, as well as the supplementary policy of a “green credit card.” In “Literature review” section, we review previous studies regarding eco-labels and purchasing behavior among consumers. In “Model” section, we describe our model and analytical methods, and in “Results and discussion” section we present our results and discuss the implications of the findings.

## Eco-labels in Korea

South Korea has rapidly industrialized and faced with environment and energy issues. For sustainable development, two types of representative eco-label have been used in South Korea since 1992: Energy Efficiency Grade Label and Korea eco-label.

Energy Efficiency Grade Label is a mandatory program operated by the Korea Energy Management Corporation (KEMCO) and is supervised under the Ministry of Trade, Industry and Energy (MOTIE) (KEMCO 2014). This label indicates energy consumption, which increases from grade 1, the lowest, to grade 5, the highest. Products that fail to meet the Energy Efficiency Grade Label requirements are banned from sale. The criteria for this label are continuously revised; e.g., CO2 emissions and annual energy expenses have recently been added. As of 2014, 24 types of products are covered by this program. Even though it is an eco-label, Energy Efficiency Grade Label also provides privately beneficial information (KEMCO 2014).

In contrast with Energy Efficiency Grade Label, Korea eco-label is a voluntary program operated by the Korea Environmental Industry and Technology Institute (KEITI) and supervised by the Ministry of Environment (MOE) (KEITI 2014). Korea eco-label is used for life-cycle assessment of products. It indicates whether products were manufactured with a low emission level of environmental pollutants or with conservation of resources. As of 2012, a total of 3030 companies are participating in the Korea eco-label program (KEITI 2014), which covers 9140 products in 155 item categories.

To provide a financial incentive for consumers to purchase Korea eco-labeled products, an eco-mileage card was launched in January 2011. It was developed as a credit card, the “green credit card”, through the cooperation of nine banks and credit card companies. Green credit card users earn economic rewards, termed ‘eco-money,’ when they purchase Korea eco-labeled products. Cardholders are also able to use public facilities such as national parks and museums free of charge or at a discounted price (KEITI 2014).

## Literature review

The aim of providing specific eco-information in eco-labels is to promote the purchase of eco-friendly products (Truffer et al. 2001). Therefore, the effect of various factors of consumers and that of eco-labels on their consumption have been analyzed (Bleda and Valente 2009; Daugbjerg et al. 2014; López-Mosquera et al. 2015; Meyer 2015; van Amstel et al. 2008; Wan Rashid et al. 2016).

Most studies on consumers have mainly focused on the role of environmental knowledge and consumers’ social-demographic factors. Meyer (2015) has investigated the role of education on consumers’ eco-friendly behavior. After changes in compulsory education in central Europe, the effect of an increased education level on eco-friendly behavior are analyzed using Eurobarometer data. This research showed that education enables people to be more concerned about social welfare, which results in eco-friendly behavior. López-Mosquera et al. (2015) presented similar results in their study of consumers’ socio-demographic factors and eco-friendly behavior in Spain, using survey data. They show that consumers’ concerns about the environment, rather than their age, gender, and income, shape their eco-friendly behavior.

A recent study on the role of consumers’ environmental knowledge emphasizes label-specific knowledge (Daugbjerg et al. 2014; Wan Rashid et al. 2016). Wan Rashid et al. (2016) compared general environmental knowledge and label-specific knowledge using survey data and found that label-related knowledge is more effective in inducing consumers’ purchasing behavior than general environmental knowledge is. Similarly, Daugbjerg et al. (2014) observed that eco-label knowledge increases consumers’ trust in eco-labels and is likely to induce their’ purchasing behavior. Studies on consumers conclude that an increase of environmental knowledge will increase their concerns about the environment and result in eco-friendly behavior, including eco-friendly consumption.

Another issue that is discussed in studies on eco-labels is eco-label design (Bleda and Valente 2009; van Amstel et al. 2008). Bleda and Valente (2009) argue that a graded eco-label is more effective than a single-level eco-label, while van Amstel et al. (2008) compared five types of eco-labels to clarify which provided more eco-information to consumers.

However, despite efforts to educate and increase awareness of eco-labels among consumers and effectively designing eco-label, the effect of the eco-label on purchasing behavior remains unclear. Some researchers insist that awareness of the eco-label does not affect purchasing behavior in relation to eco-labeled products (Horne 2009; Leire and Thidell 2005). Thøgersen (2000) studied consumers’ awareness of the eco-label and purchasing behavior using survey data from the European Union and found that only eco-friendly consumers considered the eco-label in their purchasing decisions. D’Souza et al. (2006) conducted a similar study using survey data from Australia and found no relationship between eco-labeling and purchasing behavior among general consumers.

Previous studies have not sufficiently investigated general consumer behavior in order to see why eco-labels fail to influence their purchasing behavior. To promote a certain sector of the market, it is essential to assess the purchasing behavior of general consumers (Banerjee and Duflo 2008). This also holds true for the eco-labeled products market. This market will grow when general consumers purchase eco-labeled products.

Recently, debates focusing on eco-labels and general consumers’ purchasing behavior have emerged in two fields of literature. The First is effective communication strategies for climate change messaging (Grinstein and Riefler 2015; Li et al. 2011; O’Neill and Nicholson-Cole 2009; Scannell and Gifford 2013; Smith and Petty 1996). This insists that simply delivering eco-information to consumers via eco-labels is not sufficient in influencing their purchasing behavior. There is a gap between awareness of eco-information and eco-friendly behavior.

To change consumers’ behavior, strategically framed messaging is required. Two kinds of strategies have been suggested; negative message framing versus positive message framing, local message framing versus global message framing. Negative message framing emphasizes loss while the positive message framing stresses on gains (O’Neill and Nicholson-Cole 2009; Smith and Petty 1996). Local message framing highlights the impact on residence region while Global message framing emphasizes the impact on the world (Grinstein and Riefler 2015; Scannell and Gifford 2013).

In general, consumers perceive climate change as important but not personally relevant. It is abstract and complex concept for general consumers (Grinstein and Riefler 2015). Psychologically, the lack of immediacy is one of the reasons why climate change messages fail to influence general consumers’ behavior. However, consumers accept locally framed messages more easily and immediately (Kates and Wilbanks 2003; Li et al. 2011).

The power of locally framed messages is reported in multiple studies. Scannell and Gifford (2013) analyzed the effectiveness of global and locally framed messages and found that there is greater engagement in climate change when the message delivered is about the impact on local areas. Grinstein and Riefler (2015) analyzed the effectiveness of global and local framing messages in Israel, and categorized consumers into three groups; high-, middle- and low-cosmopolitan consumers. High-cosmopolitan consumers are interested in global issues and willingly join the global movement. Global framing message is more effective on high-cosmopolitan consumers than low-cosmopolitan consumers. On low-cosmopolitan consumers, namely general consumers, local framing messages are effective because consumers can easily evaluate the impact of their purchasing behavior (Grinstein and Riefler 2015).

Another field of literature that focuses on eco-labels and purchasing behavior of consumers has emerged in relation to the private value of eco-label. Regarding the purchasing behavior of general consumers, although public value (e.g., the values provided by eco-labels) is important, the same is true of private value. Eco-friendly products have the character of impure public goods (Kotchen 2005). The concept of impure public goods suggests that products have both private and public characteristics. Grolleau et al. (2009) have theoretically analyzed the private and public nature of eco-labeled goods. They emphasized that although the public nature of eco-labeled products is important, it is not sufficient to bring consumers’ purchasing decisions. Kaufman (2014) compared the effect of financial incentive and informational campaign using simulation and found that the financial issues are more powerful in encouraging consumers purchasing behavior.

The private nature is an important incentive in terms of purchasing behaviors among general consumers. However, their study did not investigate this issue in terms of the eco-label. In this study, we applied two-dimensional (private and public) values, the concept of Grolleau et al. (2009) to the eco-label and empirically investigated general consumers’ preference of eco-label in terms of these two values in case of Korea.

## Model

We investigated the value of eco-label most preferred by consumers, in two stages. In the first stage, we use a structural equation model (SEM) to analyze the path from the perceived value of eco-label to the intention to purchase. In the second stage, the preferred value of each eco-label is investigated separately, based on the results from the first stage.

The objective of this paper was to analyze the preferred value of eco-label among consumers in terms of psychology. Accordingly, we constructed a model based on the theory of reasoned action (TRA), a social psychology theory that has been used to investigate the intentions of individuals (Conner et al. 2001). The model is widely used in various fields to analyze social behavior; e.g., Mishra et al. (2014) used TRA to analyze consumer acceptance of green information technology.

In TRA, behavior intention is defined as the intention of an individual to either perform or not perform a behavior, as the most important determinant of how a person acts (Kotchen 2005). In the present paper, purchasing intention (PI) represents the intention of the consumer to consider an eco-label in their purchasing behavior. Purchasing intention is itself influenced by the medium of attitudes, by which an individual’s beliefs about an object are translated into the intention of behavior (Schwartz 1992). Label attitude (LA) is defined as the attitude of the consumer towards eco-labels. It indicates whether the consumer searches for, reads, and gains awareness from the information provided by an eco-label.

**Fig. 1:**
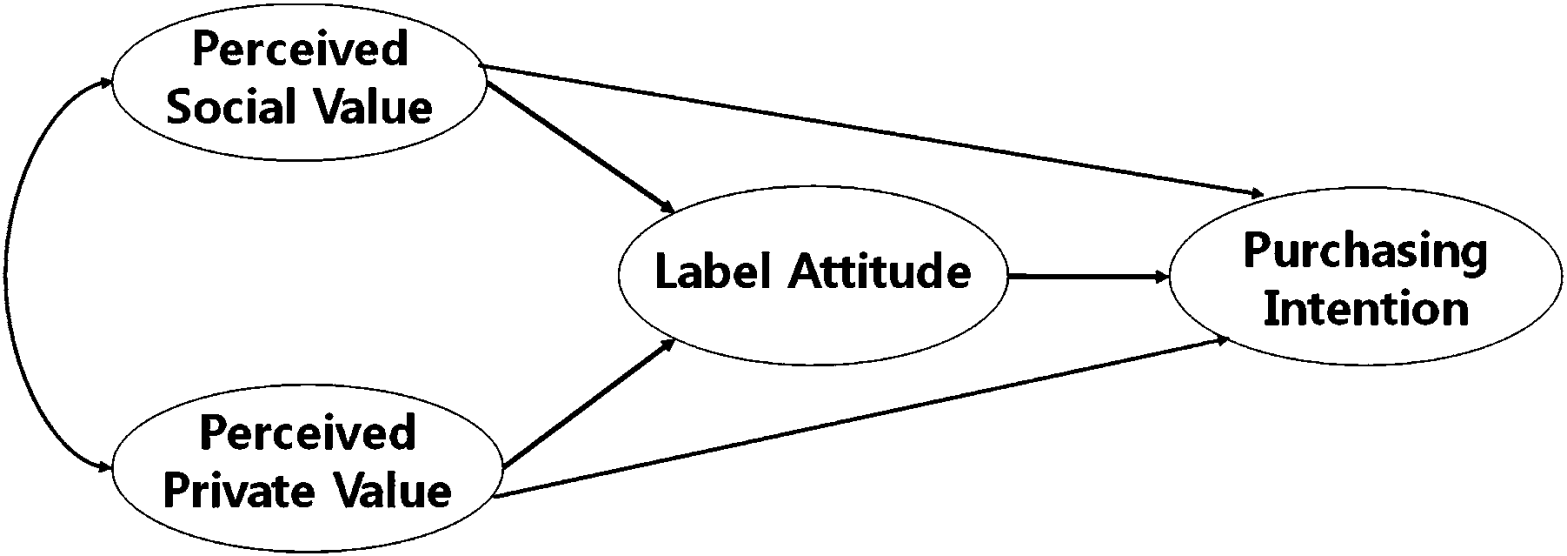
Our proposed model for preferred value of eco-label and purchasing decisions among consumers

Attitude is in turn influenced by perception (Cherian and Jacob 2012), which refers to a certain belief that consumers have about a product (Schwartz 1992). This belief consists of two dimensions, which in the present paper are termed the perceive social value (SV) and the perceived private value (PV). Grolleau et al. (2009) explained that eco-products have both social and private value. social value refers to the belief that a social benefit such as a reduction in pollution or in the use of hazardous materials will be gained through the use of a certain item, whereas private value refers to the belief that private benefit such as a saving in cost or an increase in quality of life will be gained through the use of a certain item (Grolleau et al. 2009). In the present paper, SV refers to the belief among consumers that the eco-label provides valuable social information, whereas PV refers to the belief among consumers that the eco-label provides valuable private information. The consumer can decide to purchase an eco-labeled product after reading the information provided by the eco-label; in purchasing products, they may also consider the eco-label itself because they believe that the information provided has value. Thus, in the present study we aimed to construct the paths from SV and PV to PI (Fig. 1).

In the first stage, the paths from the perceived social value and the perceived private value to the purchasing intention are estimated by the SEM. As one of the most widely used multivariate statistical tools, the SEM combines the conventional statistical analysis methods of confirmatory factor analysis (CFA), multiple regression, and path analysis to explore the entire set of relationships among the latent constructs that are indicated by multiple measures defining a study (Goldberger 1972; Hair et al. 2006). The SEM facilitates the simultaneous estimation of a series of dependent relationships, enables calculation of the measurement error in the estimation process, and is particularly effective in explaining the causality of the purchasing decision-making process (Priester 2010). It is used in various fields, including education and behavioral science (Parhizgari and Gilbert 2004).

In the second stage, we analyze the path from the social and private value to the purchasing decision, based on the results from the first stage, for each of the two types of eco-label. Energy Efficiency Grade Label provides information regarding the annual energy cost to be paid by the consumer from using the product, which is private value. In contrast, Korea Eco-Label provides eco-information in relation to life-cycle assessment, which is social value. In Korea, green credit cards provide a financial incentive for the consumer to purchase eco-labeled products, thus converting social value into private value. Accordingly, in the second stage, we investigate the consumer response to each eco-label.

### Data

To determine consumer preference among the three different types of eco-label (Energy Efficiency Grade Label, Korea Eco-Label without a green credit card, and Korea Eco-Label with a green credit card) we designed a questionnaire with 12 questions grouped under four categories in accordance with our SEM model. For the category SV, respondents were asked whether the eco-label provides adequate information regarding climate change mitigation, reduction in energy consumption, and reduction in environmental pollutants (e.g., chemicals harmful to the ozone layer). For the category PV, respondents were asked whether the eco-label provides adequate information regarding economic benefit, usefulness (e.g., safety), and ease of use (Grolleau et al. 2009). For the category LA, respondents were asked whether the eco-label provides adequate information regarding the usefulness and reliability of eco-label information, and whether the information on the eco-label is of interest to them. For the category PI, respondents were asked whether they would be willing to purchase, re-purchase or recommend eco-labeled products.

The respondents evaluated each of the 12 questions using a 7-point Likert-type scale (from 1 “not valuable” to 7 “extremely valuable”). The middle-income consumer is important to the economic success of the (12) (Banerjee and Duflo 2008), and the primary use of eco-labeling is on appliances, which are mostly purchased by adults. Therefore, the survey was conducted on middle-income adults aged ≥25 years. The questionnaire was conducted by e-mail during 26–30 November 2012, and was administered by a professional survey company. The total number of respondents was 200. Four responses were excluded from analysis due to incomprehensible data. A total of 588 responses were analyzed in Stage 1 and 196 in Stage 2.

The demographic characteristics of the respondents are summarized in Table 1. Regarding age, 25% of the respondents were aged 25–34, 33% were 35–44, 29% were 45–54, and 14% were 55 years or older. Regarding education level, 18% had a high school education and 82% had a college education or higher. The middle income group, which plays an important role in specific markets and is the target population of the present study, was defined as individuals earning 2–5 million KRW, which is 50–150% of the Korean median monthly household income (Atkinson et al. 1995) of three million KRW in 2012 (KOSTAT 2014). Of the respondents, 45% had a monthly income of two to three million KRW, 31% had a monthly income of 3–4 million KRW, and 24% had a monthly income of 4–5 million KRW.

**Table 1:** Respondents’ demographic characteristics

Group	Number of respondents (ratio)
Gender	
Male	104 (53%)
Female	92 (47%)
Age, years	
25–34	49 (25%)
35–44	64 (33%)
45–54	56 (29%)
≥ 55	27 (14%)
Education level	
High school	36 (18%)
College	160 (82%)
Monthly household income	
KRW 2–3 million	89 (45%)
KRW 3–4 million	60 (31%)
KRW 4–5 million	196 (24%)

## Results and discussion

### Result 1: Integration model

Before SEM analysis, we carried out CFA to assess the reliability and validity of the observed and latent variables (Hair et al. 2006). Cronbach’s alpha indicates the reliability of latent variables. A value over 0.7 indicates goods reliability. As shown in Table 2, Cronbach’s alpha for all latent variables was over 0.7. Convergent validity, which refers to the extent to which a set of measured variables reflects the latent construct, is verified by the standardized factor loading of the observed variables, average variance extracted (AVE), and construct reliability (CR). For good convergent validity, the standardized factor loading of the observed variables should be 0.5 or higher (Fornell and Larcker 1981). AVE and CR should be higher than 0.5 and 0.7, respectively, to indicate adequate convergent validity (Fornell and Larcker 1981; Hair et al. 2006). As shown in Table 2, the factor loadings of all observed variables exceed 0.5 and are significant at the 1% level. The AVE and CR calculations indicate acceptable convergent validity.

**Table 2:** Confirmatory factor analysis results for reliability and construct validity

Latent variables	Cronbach’s alpha	CR	AVE	Std. factor loading	SE	p-value
Perceived social value (SV)	0.95	0.95	0.87			
Climate change mitigation (SV1)				0.93		0.00
Reduction in energy consumption (SV2)				0.96	0.026	0.00
Reduction in environmental pollutants (SV3) (e.g., chemicals harmful to the ozone layer)				0.90	0.032	0.00
Perceived private value (PV)	0.92	0.92	0.80			
Economic benefit (PV1)				0.90		0.00
Usefulness (PV2) (e.g., safety)				0.93	0.037	0.00
Ease of use (PV3)				0.86	0.044	0.00
Label Attitude (LA)	0.92	0.89	0.74			
Interest of eco-label information (LA1)				0.88		0.00
Usefulness of eco-label information (LA2)				0.92	0.038	0.00
Reliability of eco-label information (LA3)				0.78	0.047	0.00
Purchasing intention (PI)	0.89	0.91	0.78			
Purchasing (PI1)				0.92		0.00
Replacement (PI2)				0.84	0.040	0.00
Recommendations (PI3)				0.88	0.038	0.00

Next, the fit of the proposed model was assessed using the Chi square (χ^2^) test, the Comparative Fit Index (CFI), and the Root Mean Square Error of Approximation (RMSEA). The results were as follows: χ^2^ = 207.022 (p < 0.001), RMSEA = 0.075, and CFI = 0.978. All values of the goodness-of-fit indices exceeded the recommended values. A standardized maximum likelihood method was used to determine path loading in the model. The standardized path loadings and their statistical significance are listed in Table 3.

**Table 3:** Standardized path loadings for each eco-label among consumers

	Path loading	SE	CR	p-value
Perceived social value (SV) → label attitude (LA)	0.29	0.055	6.219	0.00
Perceived private value (PV) → label attitude (LA)	0.57	0.056	11.671	0.00
Perceived social value (SV) → purchasing intention (PI)	0.06	0.062	1.287	0.156
Perceived private value (PV) → purchasing intention (PI)	0.37	0.053	7.742	0.00
Label attitude (LA) → purchasing intention (PI)	0.55	0.052	17.516	0.00

The results listed in Table 3 indicate that the path loading from SV to LA was 0.29, while that from PV to LA was 0.57; both path loadings were significant. The path loading from PV to LA was higher than that from SV to LA. PV was shown to affect purchasing intention. The path loading from PV to PI was 0.37, which is significant at the 1% level. This result suggests that some consumers feel as though the presence of an eco-label guarantees that a product is economically beneficial, not after acquiring some information by reading eco-labels. Furthermore, the path loading from PV to PI was larger than that from SV to LA.

The path loading from LA to PI was 0.55, which was significant at the 1% level. This result reveals that consumers’ perceived private value is more effective than social value in inducing consumers’ purchasing intention. General consumers interested in obtaining personal benefits from the eco-label in making their purchasing decision. It supports Grolleau et al. (2009)’s assertion that general consumers prefer private value over public value.

The weak effect of consumers’ perceived social value seems to be caused by the lack of message framing strategy. Consumers’ perceived social value of eco-labels is higher than private value. However, it does not effectively influence their label attitude and purchasing intention. Grinstein and Riefler (2015) and Scannell and Gifford (2013) insist that psychology distance is an important factor that shape consumers’ purchasing behavior. Korea eco-label provides information on CO2 emission during the production process. It is a global framing message. Thus, consumers do not actively response to eco-label because they are not personally relevant. If eco-labels are turned into locally framed messages, consumers’ perceived social value could be more effective in changing their attitude towards eco-labels and persuading their purchasing intention of eco-labeled products.

### Result 2: Preferred value of each eco-label

Using the standardized factor loading shown in Table 2 and the path loading shown in Table 3, we investigated SV and PV of both labels and the consumers’ response to each eco-label. SV and PV of Korea Eco-Label and Energy Efficiency Grade Label were calculated as follows;

1 $$ Social\,Value = \sum\limits_{i = 1}^{3} {\left( {Factor\,loading\,_{svi} \cdot \overline{{SV_{i} }} } \right)} $$

2 $$ Private\,Value = \sum\limits_{i = 1}^{3} {\left( {Factor\,loading_{pvi} \cdot \overline{PV}_{i} } \right)} $$

The results are presented in Table 4.

**Table 4:** Preferred value of each eco-label among consumers

	Energy efficiency grade label	Korea eco-Label (without green card)	Korea eco-label (with green card)
Perceived social value (SV)	16.15	15.34	14.77
Perceived private value (PV)	15.99	12.86	13.19
Perceived social value (SV) → label attitude (LA)	4.68	4.45	4.28
Perceived private value (PV) → label attitude (LA)	9.11	7.33	7.52
Label attitude (LA)	13.80	11.78	11.80
Perceived private value (PV) → purchasing intention (PI)	5.92	4.76	4.88
Label attitude (LA) → purchasing intention (PI)	7.59	6.48	6.49
Purchasing intention (PI)	13.50	11.24	11.37

The SV and PV of Energy Efficiency Grade Label were 16.15 and 15.99, respectively. SV and PV of Korea Eco-Label without a green card were 15.34 and 12.86, respectively. With a green card, SV and PV of Korea Eco-Label were 14.77 and 13.19, respectively. The results revealed that in all cases, SV was larger than PV. However, the path loading from SV to LA was smaller than that from PV to LA in all cases. Regarding Energy Efficiency Grade Label, the path loading from SV to LA was 4.68, while that from PV to LA was 9.11. Regarding Korea Eco-Label without a green card, the path loading from SV to LA was 4.45, but that from PV to LA was 7.33. Regarding Korea Eco-Label with a green card, SV to LA was 4.28, but perceived private value to label attitude was 7.52. These results suggest that although consumers are aware of the public benefits of eco-labeled products, this awareness does not promote a positive attitude. In contrast, PV of eco-label had a more marked effect on LA. In addition, the path loading from PV to PI was 5.92, 4.76, and 4.88 for Energy Efficiency Grade Label, Korea Eco-Label without, and Korea Eco-Label with a green card, respectively. Therefore, the total effect of PV on PI was high. The path from LA to PI was 7.59, 6.48, and 6.49 for Energy Efficiency Grade Label, Korea Eco-Label without, and Korea Eco-Label with a green card, respectively.

The stronger effect of PV appears much clearer when the results of each label are examined in detail. Energy Efficiency Grade Label was more effective than Korea Eco-Label on PI. Part of the gap between Energy Efficiency Grade Label and Korea Eco-Label was observed in the path from PV to LA. The PV gap between Energy Efficiency Grade Label and Korea Eco-Label was 3.13 without and 2.8 with a green card, which was relatively high compared with the SV gap between both labels. The SV gap between Energy Efficiency Grade Label and Korea Eco-Label was 0.81 without and 1.38 with a green card. The larger PV gap between Energy Efficiency Grade Label and Korea Eco-Label resulted in a larger gap in the path to LA.

Both Energy Efficiency Grade Label and Korea Eco-Label have been used since 1992. A total of 24 types of product are covered by Energy Efficiency Grade Label, which is only about half the number of items covered by Korea Eco-Label. However, our results suggest that Energy Efficiency Grade Label had a greater effect on PI. This indicates that general consumers consider eco-labeling in their PI because the eco-label is more privately valuable. Furthermore, Energy Efficiency Grade Label provides information on energy costs that must be paid by consumers.

Regarding Korea Eco-Label, after the introduction of the green credit card, the SV decreased while the PV increased. The decrease in SV was greater than the increase in PV. It therefore appears that the green credit card converted SV to PV by providing a financial incentive to purchase eco-labeled products. However, the changes observed in path loadings from both PV and SV to LA were similar. The path loading from SV to LA decreased by 0.17, whereas that from PV to LA increased by 0.18. However, the increase in PV affected the path loading towards PI. As a result, PI increased by 0.13.

These results suggest that although people are aware of the SV of eco-labels, this awareness does not affect their purchasing behavior. Furthermore, although general consumers are aware that some products are eco-friendly goods, their purchasing behavior is influenced more by PV. Mitomo and Otsuka (2012) explained that because the environment degrades slowly, consumers tend not to take it seriously and rarely consider the eco-information.

## Conclusion

The purpose of eco-labels is to provide eco-information and to increase the sale of eco-labeled products. In some research, consumers have expressed a higher willingness to pay for eco-labeled products. However, in the real world, the introduction of eco-labels has not encouraged consumers to purchase eco-labeled products. A gap is evident between eco-label awareness and purchasing behavior in relation to eco-labeled products. To increase the market for eco-labeled products, it is essential that eco-labeled products be purchased by general consumers.

The results from this study reveal that consumers’ perceived private value is more effective than their perceived social value. It has two implications. Firstly, a strategy for consumers’ private value is required. When an eco-label indicates that a product allows consumers to save money or cut costs, it will be purchased. If no private value is provided by an eco-label, the introduction of an alternative method, such as a cash-back system in which consumers can convert prior purchases to cash, could be a solution to encourage the purchase of eco-labeled products.

Secondly, a strategy for consumers’ perceived social value is required. Consumers are highly aware of the social value of eco-labels. They perceive social value as higher than private value. However, this does not effectively induce consumers’ attitude and purchasing intention. Local framing message strategy could be a solution. Research on message framing strategy suggests that locally framed messages encourage consumers’ purchasing behavior (Grinstein and Riefler 2015; Scannell and Gifford 2013). Consumers are more likely to accept local framed messages as personally relevant information. They can easily evaluate the information and immediately respond to it.

South Korea has been faced with environmental and energy issues and announced ‘Green Growth’ as a national strategy. Consumers are becoming highly aware of the importance of purchasing eco-labeled products. Therefore the results in this paper will be the lesson to other developing countries. To ensure sustainable development, purchasing eco-friendly products needs to be more advantageous than purchasing non-eco-friendly products. Policy makers believe that eco-friendly products become popular when consumers receive eco-information in relation to those products. However, consumers respond to eco-labels only when they receive private benefits. Therefore, policy makers should consider how to convert the public value associated with eco-labels to private value associated with the consumer.

## References

[CR1] Atkinson AB, Rainwater L, Smeeding TM (1995) Income distribution in OECD countries: evidence from the Luxembourg Income Study. Organisation for Economic Co-operation and Development (OECD), Paris

[CR2] Banerjee AV, Duflo E (2008). What is middle class about the middle classes around the world?. J Econ Perspect.

[CR3] Bleda M, Valente M (2009). Graded eco-labels: a demand-oriented approach to reduce pollution. Technol Forecasti Soc.

[CR4] Cherian J, Jacob J (2012). Green marketing: a study of consumers’ attitude towards environment friendly products. Asían Soc Sci.

[CR5] Conner M, Kirk SFL, Cade JE, Barrett JH (2001). Why do women use dietary supplements? The use of the theory of planned behaviour to explore beliefs about their use. Soc Sci Med.

[CR6] Daugbjerg C, Smed S, Andersen LM, Schvartzman Y (2014). Improving eco-labelling as an environmental policy instrument: knowledge, trust and organic consumption. J Environ Policy Plan.

[CR7] D’Souza C, Taghian M, Lamb P (2006). An empirical study on the influence of environmental labels on consumers. Corp Commun.

[CR8] Fornell C, Larcker DF (1981). Evaluating structural equation models with unobservable variables and measurement error. J Mark Res.

[CR9] Goldberger AS (1972). Structural equation methods in the social sciences. Econometrica.

[CR10] Grinstein A, Riefler P (2015). Citizens of the (green) world? Cosmopolitan orientation and sustainability. J Int Bus Stud.

[CR11] Grolleau G, Ibanez L, Mzoughi N (2009). Too much of a good thing? Why altruism can harm the environment?. Ecol Econ.

[CR12] Hair JF, Black WC, Babin BJ, Anderson RE, Tatham RL (2006). Multivariate data analysis.

[CR13] Horne RE (2009). Limits to labels: the role of eco-labels in the assessment of product sustainability and routes to sustainable consumption. Int J Consum Stud.

[CR14] Kates RW, Wilbanks TJ (2003). Making the global local responding to climate change concerns from the ground. Environ Sci Policy Sustain Dev.

[CR15] Kaufman N (2014). Overcoming the barriers to the market performance of green consumer goods. Resour Energy Econ.

[CR16] KEITI (2014). Policy handbook for sustainable consumption and production of Korea.

[CR17] KEMCO (2014). Annual report 2013.

[CR18] KOSTAT (2014). Statistics of households’ financial and welfare.

[CR19] Kotchen MJ (2005). Impure public goods and the comparative statics of environmentally friendly consumption. J Environ Econ Manag.

[CR20] Leire C, Thidell Å (2005). Product-related environmental information to guide consumer purchases—a review and analysis of research on perceptions, understanding and use among Nordic consumers. J Clean Prod.

[CR21] Li Y, Johnson EJ, Zaval L (2011). Local warming: daily temperature change influences belief in global warming. Psychol Sci.

[CR22] López-Mosquera N, Lera-López F, Sánchez M (2015). Key factors to explain recycling, car use and environmentally responsible purchase behaviors: a comparative perspective. Resour Conserv Recycl.

[CR23] Meyer A (2015). Does education increase pro-environmental behavior? Evidence from Europe. Ecol Econ.

[CR24] Mishra D, Akman I, Mishra A (2014). Theory of reasoned action application for green information technology acceptance. Comput Human Behav.

[CR25] Mitomo H, Otsuka T (2012). Rich information on environmental issues and the poor reflections on consumers’ green actions: a behavioral economic approach. Telemat Inform.

[CR26] O’Neill S, Nicholson-Cole S (2009). “Fear won’t do it”: promoting positive engagement with climate change through visual and iconic representations. Sci Commun.

[CR27] Parhizgari A, Gilbert GR (2004). Measures of organizational effectiveness: private and public sector performance. Omega.

[CR28] Priester JR (2010). The use of structural equation models in consumer psychology: a methodological dialogue on its contributions, cautions, and concerns. J Consum Psychol.

[CR30] Rex E, Baumann H (2007). Beyond ecolabels: what green marketing can learn from conventional marketing. J Clean Prod.

[CR31] Scannell L, Gifford R (2013). Personally relevant climate change: the role of place attachment and local versus global message framing in engagement. Environ Behav.

[CR32] Schwartz SH, Mark PZ (1992). Universals in the content and structure of values: theoretical advances and empirical tests in 20 countries. Advances in experimental social psychology.

[CR33] Smith SM, Petty RE (1996). Message framing and persuasion: a message processing analysis. Pers Soc Psychol B.

[CR34] Thøgersen J (2000). Psychological determinants of paying attention to eco-labels in purchase decisions: model development and multinational validation. J Consum Policy.

[CR35] Truffer B, Markard J, Wüstenhagen R (2001). Eco-labeling of electricity—strategies and tradeoffs in the definition of environmental standards. Energ Policy.

[CR36] van Amstel M, Driessen P, Glasbergen P (2008). Eco-labeling and information asymmetry: a comparison of five eco-labels in the Netherlands. J Clean Prod.

[CR37] Wan Rashid WE, Muda M, Taufique KMR, Siwar C, Chamhuri N, Sarah FH (2016). The fifth international conference on marketing and retailing (5th INCOMaR) 2015 integrating general environmental knowledge and eco-label knowledge in understanding ecologically conscious consumer behavior. Procedia Econ Finance.

